# Between-Site Differences in the Scale of Dispersal and Gene Flow in Red Oak

**DOI:** 10.1371/journal.pone.0036492

**Published:** 2012-05-01

**Authors:** Emily V. Moran, James S. Clark

**Affiliations:** 1 National Institute for Mathematical and Biological Synthesis, University of Tennessee, Knoxville, Tennessee, United States of America; 2 Nicholas School of the Environment, Duke University, Durham, North Carolina, United States of America; University of Western Ontario, Canada

## Abstract

**Background:**

Nut-bearing trees, including oaks (*Quercu*s spp.), are considered to be highly dispersal limited, leading to concerns about their ability to colonize new sites or migrate in response to climate change. However, estimating seed dispersal is challenging in species that are secondarily dispersed by animals, and differences in disperser abundance or behavior could lead to large spatio-temporal variation in dispersal ability. Parentage and dispersal analyses combining genetic and ecological data provide accurate estimates of current dispersal, while spatial genetic structure (SGS) can shed light on past patterns of dispersal and establishment.

**Methodology and Principal Findings:**

In this study, we estimate seed and pollen dispersal and parentage for two mixed-species red oak populations using a hierarchical Bayesian approach. We compare these results to those of a genetic ML parentage model. We also test whether observed patterns of SGS in three size cohorts are consistent with known site history and current dispersal patterns. We find that, while pollen dispersal is extensive at both sites, the scale of seed dispersal differs substantially. Parentage results differ between models due to additional data included in Bayesian model and differing genotyping error assumptions, but both indicate between-site dispersal differences. Patterns of SGS in large adults, small adults, and seedlings are consistent with known site history (farmed vs. selectively harvested), and with long-term differences in seed dispersal. This difference is consistent with predator/disperser satiation due to higher acorn production at the low-dispersal site. While this site-to-site variation results in substantial differences in asymptotic spread rates, dispersal for both sites is substantially lower than required to track latitudinal temperature shifts.

**Conclusions:**

Animal-dispersed trees can exhibit considerable spatial variation in seed dispersal, although patterns may be surprisingly constant over time. However, even under favorable conditions, migration in heavy-seeded species is likely to lag contemporary climate change.

## Introduction

Nut-bearing trees, including oaks (*Quercus*), beech (*Fagus*), walnut (*Juglans*), and hickory (*Carya*), are ecologically and economically important components of many temperate forests [1]–[3]. These species produce relatively small numbers of heavy, animal-dispersed seed [4], [5], so dispersal limitation may hinder their ability to colonize new sites or respond to climate change through range shifts [6]–[9]. Nevertheless, variation in the availability and behavior of dispersers is likely to lead to spatial and temporal variability in dispersal ability [10]–[14]. Because nuts are buried by animal vectors [5], seed trap data are insufficient to capture the full dispersal kernel. However, genetic data are beginning to shed light on dispersal processes in the Fagaceae (*Quercus*, *Fagus*, *Castanea*) [8], [15]–[19], as well as in many other plant taxa [20]. Two approaches have been used to infer dispersal from genetic data: a) Parentage and dispersal analyses, which reveal current patterns of gene flow, and b) spatial genetic structure (SGS) analyses, which reflect historical patterns of dispersal and establishment. To date, relatively few genetic studies of trees have estimated full dispersal kernels at more than one site and/or paired contemporary dispersal estimates with SGS analyses [8], [15], [21]–[24]. In this study, we estimate seed and pollen dispersal and parentage for two mixed-species red oak populations (*Q. rubra*, *Q. velutina*, *Q. falcata*, *Q. coccinea*) using a new hierarchical Bayesian approach [25], and investigate whether the observed differences are consistent with differences in disperser abundance or stand structure. We then test whether observed patterns of SGS in three cohorts (large adults, small adults, and seedlings) are consistent with known site history and current dispersal patterns. We also compare results for the Bayesian model to a purely genetic maximum likelihood model (CERVUS) [26]. Only one previous study of forest trees has combined genetic estimation of dispersal kernels with SGS analysis at multiple sites [8]; ours is the first such study in oaks, and the first to use a Bayesian approach. Finally, we discuss the implications of variation in dispersal ability for range-shifts.

In plants, the scale of seed dispersal strongly influences the ability of the species to colonize new areas [9], [27], [28], while gene flow via both seed and pollen has important implications for local adaptation and the maintenance of genetic diversity [29], [30]. Quantifying seed and pollen dispersal in many forest trees is complicated by the cryptic nature of these processes [4]. Pollen dispersal by wind is impossible to observe directly, and physical pollen transport distances can differ markedly from effective pollen dispersal distances [31], [32]. The seeds of nut-bearing trees are dispersed by scatterhoarding animals that bury seeds in shallow caches [5]; The seed-trap data typically used to estimate seed dispersal kernels do not include such secondary dispersal. Genetic markers are useful in reconstructing pedigrees and estimating both seed and pollen movement [20], provided that models can account for the complexity of pollen dispersal from father to mother, seed dispersal from mother to the location of offspring, incomplete genotyping, and the existence of genotyping errors [25].

Dispersal, together with factors affecting establishment, creates the spatial genetic structure of populations. Inbreeding and restricted seed dispersal generate positive genetic autocorrelation, while long-distance dispersal tends to reduce SGS [33]. In order to infer the scale of past dispersal from SGS, it is necessary to take mortality and the history of a site into account. When recruits derive from widely scattered seed sources, SGS is weak to non-existent [34]. SGS tends to increase in subsequent generations due to local recruitment and bi-parental inbreeding [35]–[37], until overlapping seed shadows once again reduce spatial correlation in genotype.

Many genetic studies of dispersal in forest trees have described the distribution of observed mother-offspring distances [16]–[19], [23], [38]–[41], but relatively few have estimated full dispersal kernels. Standard parentage analyses are based solely on genetic data: potential parents are either excluded by genotypic mismatches [18] or the likelihood of parentage is calculated to allow for genotyping error [26], [42]. In plants, however, many species are hermaphroditic, seed and pollen movement is distance dependent [20], [32], and individual fecundity tends to be related to size [43], [44]; considering such factors can help to distinguish between potential parents with similar likelihoods of producing the observed offspring genotype [45]. Moreover, constructing dispersal kernels based solely on the distance to most likely parent within the mapped stand can lead to strongly biased estimates, especially when dispersal from outside the mapped stand is not considered [46]. The full probability model approach estimates dispersal parameters directly rather than deriving them from parentage results [45], [47]. The seedling neighborhood model of Burczyk et al. [47] has been used to compare seed and pollen dispersal distances for two mixed *Quercus robur*/*Q.petraea* populations [15] and three populations of *Fagus sylvatica* [8]. In the former, acorn and pollen dispersal patterns differed between sites, while in the *Fagus* study, dispersal distances were similar across sites for young seedlings. However, the neighborhood model does not account for genotyping error [15].

In a previous paper [25], we described a new hierarchical Bayesian approach to estimating parentage and dispersal parameters. This model integrates multi-locus genetic data from adults and seedlings with ecological data, allows for dispersal from outside the plot and, unlike many earlier models, accounts for two types of genotyping error. We demonstrated this model using data from a mixed-species population of red oak located in Duke Forest in the North Carolina Piedmont. A hierarchical Bayesian approach presents a number of advantages for the study of dispersal, including the capacity to accommodate multiple data types, multiple sources of uncertainty, and existing (“prior”) information with relative ease within a fully consistent framework [42], [48]. It also allows for a smooth propagation of uncertainty [49], [50] so that, for instance, the posterior distribution for a dispersal parameter reflects uncertainty in both data and parentage assignment. Our aim in this study was to investigate site-to-site variation in dispersal and the ecological factors that may lead to divergent dispersal patterns by extending that model to a second population located at Coweeta LTER in the Southern Appalachians. In order to compare contemporary and historical dispersal, we also calculated SGS for three cohorts, and developed a simulation to test whether the differences in SGS between age groups and sites were consistent with site history and with the scale of current gene flow. We hypothesized that:

1) Long-distance effective seed dispersal would be associated with a high density of dispersers.
2) Because animals often cache seed at shorter distances when seed is abundant [12], effective seed dispersal would be lower when acorn abundance is high.
3a) Because the Duke Forest site was cleared for farming, and oaks likely recruited from seed sources outside the site, SGS in the oldest cohort should be weak to non-existent. In the second cohort SGS, recruiting from local sources, SGS should be strong, while increasing seed shadow overlap should result in lower SGS in seedlings.
3b) Because the Coweeta site was selectively logged, retaining saplings, SGS in the older generation should be strong. SGS in the 2^nd^ cohort and in seedlings should be progressively weaker due to greater overlap in seed shadows.

## Materials and Methods

### Study sites

The Duke Forest site (Table 1) was cleared for agriculture prior to the 20^th^ century, though some forest patches may have been maintained as selectively-cut woodlots. Historical documents and loblolly pine [51] tree-ring data suggest the site was abandoned between 1912 and 1921. Today the tree community in the Duke Forest plot includes mature loblolly pines (*Pinus taeda*) inter-mixed with hardwoods such as *Quercus*, *Carya*, *Acer*, and *Liquidambar*. The Coweeta study site was selectively logged in the early 1900's, but stems <12 cm diameter-at-breast-height (DBH) were retained. The site has substantial topographical relief, and today is dominated by mixed hardwoods including *Quercus*, *Acer*, and *Liriodendron*, as well as *Rhododendron* thickets. Clumping of red oak seedlings at densities as high as 7.5/m^2^ near adult trees suggested that dispersal at Coweeta might be more limited than at Duke Forest. Coweeta supports a higher density of both oak seedlings and total understory vegetation than Duke Forest.

**Table 1:** Site Characteristics.

	Duke Foresthttp://www.env.duke.edu/forest	Coweetahttp://coweeta.ecology.uga.edu
Location	35°58′N; 79°5′W	35°03′ N; 83°27′W
Elevation (m)	155	1030
Area (ha)	12	7.5
Site history	Farmed until early 1900's	Selectively cut, early 1900's
**Sample Size**		
Adult red oaks	118	199
Oak seedlings	219	179
**Density**		
Adult red oaks/ha	9.8	**26.5**
Oak seedlings/m^2^	0.41	**1.27**
**Mean seed production**(2000–2008)		
Red oak acorns/ha	11,748 (5,458–15,830)	**27,518** (14,274–91,962)
**Mean recruitment**		
New seedlings per seed	0.011 (0.001–0.017)	**0.047** (0–0.154)
**Mean annual survival**(2005–2009)		
1^st^ yr seedlings	**85%**	66.7%
Older seedlings	95.9%	98.2%

### Study species

Several members of the red oak clade (section *Lobatae*) coexist at our study sites: northern red oak (*Q. rubra*), black oak (*Q. velutina*) and southern red oak (*Q. falcata*) at Duke Forest, and *Q. rubra*, *Q. velutina*, and scarlet oak (*Q. coccinea*) at Coweeta. Co-occurring species grow closely intermixed at each site. Oak species have a high ability to hybridize within sections of the genus [52]–[54]. These four species have all either been observed to hybridize [55], [56] or are known to be closely related [57]. Genetic structure analyses show very little genetic differentiation between morphologically-defined species at each site, and excluding any species from the parentage analysis results in a significant increase in the number of seedlings with no plausible parent within the plot, which suggests moderate to high levels of past and current hybridization [58]. Consequently, in the primary analyses that follow, all individuals at each site are treated as a single interbreeding population. However, we also analyzed data for each species at each site separately, to test the effect of our single-population assumption on parentage and dispersal estimates.

Oaks produce relatively few, heavy seeds, and previous dispersal studies suggest that acorn movement is often restricted [15], [18], [59], [60]; however, birds can be efficient long-distance dispersal (LDD) vectors of acorns, transporting seeds hundreds of meters to several kilometers [13], [14], [61], [62]. Grey squirrels (*Sciurus carolinensis*) and blue jays (*Cyanocitta cristata*) are the most important dispersers of oaks in southeastern oak-hickory forests [5]. LDD of pollen by wind is thought to maintain genetic connectivity over large areas [18], [63], although in dense stands effective pollen dispersal could be limited by the “swamping” of stigmas by pollen from neighboring trees [64].

### Data collection

Both sites contain an array of permanent seedling census plots. As part of earlier forest dynamics studies, 1×2 m plots (70 at Coweeta, 124 at the larger Duke Forest site) were established in cross-shaped transects [4], [65]. To increase sample size at Duke Forest, where the seedling layer is sparse, 79 1 m^2^ plots and 70 7 m^2^ census plots were added. No plot was <30 m from the edge of the mapped stand. All adult trees >10 cm DBH were considered potential parents. This was a conservative cutoff, as individuals less than 25 cm DBH are seldom reproductively mature [66]. Adult canopy leaves were obtained using a slingshot, and seedlings from the census plots were sampled non-destructively. At Duke Forest, there were 68 adult *Q. rubra*, 22 *Q. velutina*, and 28 *Q. falcata*; At Coweeta, there were 129 adult *Q. rubra*, 15 *Q.velutina*, and 54 *Q. coccinea*. Of the sampled seedlings, at Duke Forest 96 were *Q. rubra*, 85 were *Q. velutina*, and 38 were *Q. falcata*, while at Coweeta 159 were *Q. rubra*, 13 were *Q. velutina*, and 7 were *Q. coccinea*. Total sample sizes are shown in table 1. Leaf tissue was stored at −80°C prior to total genomic DNA extraction [25]. Six nuclear microsatellites isolated by Aldrich et al. [67], [68] were analyzed using GeneMarker (Softgenetics). All individuals had unique genotypes.

No specific permits were required for the described field studies. Field studies did not involve any endangered or protected species.

### Dispersal and parentage analysis

In this analysis we made use of the novel Bayesian parentage and dispersal model described in Moran and Clark [25]. This model incorporates genotypes, locations, and individual fecundities to simultaneously estimate parentage and seed and pollen dispersal parameters. As in all Bayesian models, the probability of the parameters to be estimated given the data is proportional to the probability of the data given the parameters (the likelihood) multiplied by the probability of the parameters (priors). In this case,
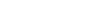


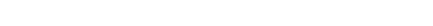


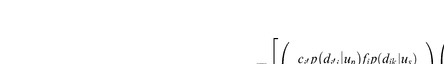
where *k* indicates the offspring, *i* the proposed mother tree, and *i′* the proposed father tree; *P* is the pedigree (mother and father for each seedling); *u_s_* and *u_p_* are seed and pollen dispersal parameters; *G^o^* is the observed genotype; *d_i′i_* is the distance between the proposed parents and *d_i′k_* is the distance between seedling *k* and tree *i*; *f_i_* and *c_i′_* are estimated seed production for *i* and pollen production for *i′*; *e_1_* and *e_2_* are mistyping and allelic dropout rates; and *l* is the locus. The first component on the left-hand side indicates that the probability of a seed dispersing to a given location depends on how far away the mother tree is and how many seeds it produces; the probability that one tree will be pollinated by another depends on how far away the father tree is and how much pollen it produces. The second component calculates the probability that two potential parents could produce an offspring with the observed genotype given their own observed genotypes and genotyping error. Finally, *p(u_s_)* and *p(u_p_)* are truncated normal prior distributions for the dispersal parameters. Priors were chosen based on estimates in the literature for seed and pollen movement in *Quercus*. The prior for *u_s_* was assigned a mean of 253, corresponding to an average distance of 25 m, and a standard deviation of 1000, truncated at values corresponding to <5 m and >157 m. The prior for *u_p_* was assigned a mean of 2000, corresponding to an average distance of 70.2 m, and a standard deviation of 1500, truncated at values corresponding to <5 m and >192 m. See the online supplement to Moran and Clark 2011 [25] for a full discussion of prior choice.

2D-t dispersal kernels [69] were fitted for both seed and pollen. For this functional form, the expected dispersal distance is equal to 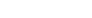. While there is currently no consensus on which functional form is most widely applicable for dispersal in plants [70], both genetic and ecological data indicate that in most tree species the distribution of seed and pollen dispersal distances is convex at the source and “fat-tailed”, with more long-distance and fewer mid-distance dispersal events than in a normal distribution [21], [71]–[74]. The 2D-t kernel meets these criteria; in addition, it allowed easier comparison to previous work done at these sites [4], [6], [69] and, with only a single parameter, can be fit with limited data. All adult trees within the stand were considered as both potential mothers and fathers of each seedling although, because selfing is rare in oaks and red oaks are believed to be self-incompatible [31], [75], we assumed that a tree could not be both mother and father to the same seedling. We did not assume that the closest parent was the mother; rather, the model mixes over uncertainty in maternity vs. paternity.

Rates of mistyping (mistaking an allele for one of similar length due to stutter in amplification) and allelic dropout (failure of one allele to amplify) were estimated for each of the 6 loci by re-genotyping many individuals [76]. Average fecundities and their standard deviations for trees within the original mapped area were calculated using a model developed by Clark et al. [4] which incorporates seedtrap and diameter-growth data to estimate the probability of maturity, and annual fecundity given maturity, for each tree. Fecundities for trees in the additional mapped area were estimated based on the fitted parameters from the Clark et al. model and their diameter, as explained in the supplement to Moran and Clark 2011 [25]. Average individual seed production per year ranged from 0 to 2,786 with a mean of 948 at Duke Forest and from 0 to 2,955 with a mean of 910 at Coweeta. We assume that pollen production is roughly proportional to seed production [25]. The probability of seed or pollen dispersal from outside the mapped stand depended on the distance of the census plot or mother tree to the edge of the stand. Because both stands were part of a continuous forest, the average density and fecundity of oaks outside was assumed to be similar to inside the mapped stand. The model was implemented in R (www.r-project.org) using a combination of Gibbs and Metropolis sampling. At each MCMC step, a fecundity value is drawn from the posterior distribution defined by the mean and standard deviation, mixing over uncertainty in fecundity; parameters were then updated using conditional probability relationships [25]. MCMC chains were run for 50,000 steps; a burn-in sequence of 30,000 steps was discarded. Posterior means and standard deviations were calculated based on every 20^th^ value of the remaining 20,000 steps. Output of the model includes posterior distributions for *u_s_* and *u_p_*, as well as for the parentage of each seedling (a 2-dimensional multinomial probability distribution). Further details can be found in the online supplement (Text S1) or Moran and Clark [25].

We compared the results of the Bayesian model under the assumption of hybridization to the genotype-only ML model CERVUS [26]. CERVUS calculates the likelihood ratio (expressed as a LOD score) for each proposed parent based on genotype, ranking parents or parent pairs according to LOD score. Besides the fact that the Bayesian model simultaneously estimates parentage and dispersal kernels, while CERVUS focuses solely on parentage, there are several other important differences between the models. First, CERVUS assumes a single genotyping error rate for all loci, and assumes that any genotyping error is equally likely (any allele can be mistaken for any other allele); the Bayesian model distinguishes between allelic dropout and mistyping (in which an allele is mistaken for one of adjacent length) and separate error probabilities are calculated for each locus using repeat genotyping data. Error rates ranged between 0.02 and 0.08 for dropout and 0.02 and 0.18 for mistyping [25], so we used an error rate of 0.09 in the CERVUS analysis. Second, CERVUS does not consider distance, whereas the Bayesian model modifies the probability of parentage depending on the distance between individuals (how much the probability is modified depends on the currently imputed value of the dispersal parameter). Third, CERVUS does not consider differences in fecundity, whereas the Bayesian model includes the assumption that a highly fecund individual will disperse more seed to a given location than a less fecund individual. Fourth, CERVUS input includes the proportion of parents genotyped, but does not distinguish between individuals within a mapped stand (all genotyped) and individuals outside the stand (all ungenotyped), as the Bayesian model does. As the exact proportion of ungenotyped parents is unknown prior to the parentage analysis, we used a genotyping percentage of 80%. Finally, CERVUS always identifies 2 in-plot parents, even if the LOD scores are low; there is no “outside” option, as there is in the Bayesian model. We compared the most-likely parent pair identified by CERVUS to the pair most frequently identified by the Bayesian model. We also plotted the distance between seedlings and the closest member of the CERVUS most likely parent pair.

### Density of potential seed dispersers

Distance-sampling based on fixed transects is an effective and cost-efficient method of estimating density for grey squirrels [77]. We established a series of transects at each site (eight, with a total length of 540 m, at Coweeta; nine, with a total length of 740 m, at the larger Duke Forest Site). On each sampling date, we recorded perpendicular distances from the transect for all potential dispersers. Following a pilot survey at Duke Forest in October 2009, survey dates were chosen such that environmental conditions (temperature, phenology) would be similar at the two sites. We conducted five surveys over two days at each site (Table S1).

Many squirrels were observed on the survey days. No blue-jay activity was observed, but previous studies indicate that jays are common at both sites. Breeding bird surveys conducted at Coweeta revealed average densities of 14.5/km^2^ in 1993 in undisturbed habitats where oaks are common [78]. Blue jays are present year-round in the Blackwood division of Duke Forest; assuming a detection distance of up to 200 m for jay calls, the average density is ∼4.46/km^2^ (www.duke.edu/~jspippen/birds/dukeforestsurvey.htm). As non-calling birds may not be detected, this is a conservative estimate.

We conducted surveys after leaf-fall, when acorns were ripe and visibility exceeded 20–50 m. All surveys occurred in the early morning or evening, when squirrels are most active [77]. Squirrels were seen actively foraging and caching acorns. Squirrel density was estimated using the program DISTANCE 6 [79]. Three functions for decay of detection probability were compared: uniform/cosine - the recommended omnibus model [80], half-normal/hermite-polynomial, and uniform/simple-polynomial. We used AIC for model selection.

### Spatial genetic structure – simulations

To better define our expectations about the impact of stand structure and dispersal scale on SGS, we conducted a series of simulations (Text S2). We defined 3 “generations”: the individuals in the first generation corresponding to the number and location of large adults, the second to small adults, and the third to seedlings. Because tree core data were only available for a small percentage of trees (34% at Duke Forest, 17% at Coweeta), dividing trees by age would have resulted in an insufficient sample size for SGS analysis. Therefore, adults were divided into large individuals (DBH>median) and small individuals (DBH<median) as a proxy for older and younger cohorts [39]. The median DBH was 33 cm at Duke Forest, 43 cm at Coweeta. By using the actual location and numbers of individuals in each cohort, we control for the effects of mortality. The simulations based on the Duke Forest site will be referred to below as “DF” and those based on Coweeta as “C”.

Given that Duke Forest site was cleared for farming, while the Coweeta was selectively logged, the most likely scenarios for oak recolonization are 1) DF, recolonization from several source populations outside the study stand (Figure S1, top left), and 2) C, regeneration from scattered source trees both within and beyond the study site (Figure S2, top middle). A density of 1.5 source trees/ha was chosen for condition 2, because this density is substantially below the current oak density at both sites (Table 1), but sufficiently high that at least 10 simulated source trees fall inside the mapped stand. We also considered scenarios in which C trees derive from 3) a lower density of source trees (0.5/ha) or 4) several source populations outside the study stand, and in which DF trees derive from 5) outside source populations plus three local source trees, 6) from sparse scattered source trees (0.5/ha), and 7) from moderately dense scattered source trees (1.5/ha).

We randomly assigned simulated source-tree parents to “first generation” trees (large adults) based on the probability of seed dispersal from each simulated source tree to the location of each 1^st^ generation tree and the probability of pollen transfer between source trees given *u_p_* = 9000 and *u_s_* = 20, 100, 800, 3500, or 7000 (Text S2). These dispersal parameters correspond to an expected pollen dispersal distance of 149 m, and expected seed dispersal distances of 7 m, 16 m, 44 m, 93 m, and 131 m. Assuming source trees are unrelated, the coefficient of relatedness of a pair of 1^st^ generation trees is 0.5 if they are full sibs, 0.25 if they are half-sibs, and zero otherwise. We calculated the average coefficient of relatedness over 100 simulations for 10 m distance classes from 0 to 100 m. For the “2^nd^ generation” (small adults), potential parents include both original source trees and 1^st^ generation trees; coefficients of relatedness can therefore take on higher values if the parents of 2^nd^ generations are themselves siblings or parent-and-child. Similarly, for the “3^rd^ generation” (seedlings), potential parents include all older cohorts.

**Figure 1:**
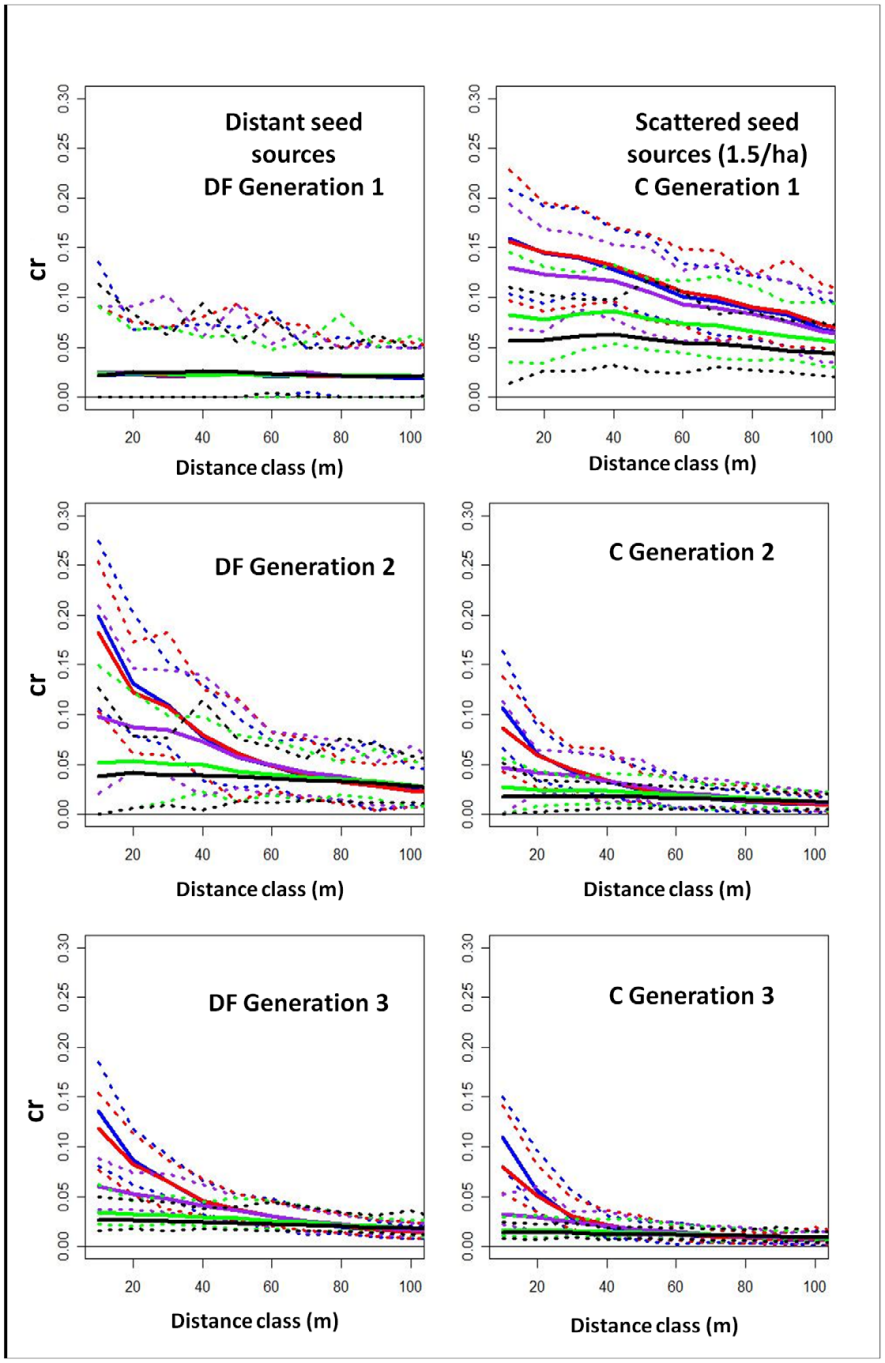
SGS simulation results. Average coefficient of relatedness (cr) at distances up to 100 m for u_s_ = 20 (blue), 100 (red), 800 (purple), 3500 (green), 7000 (black). Left: several out-of-plot seed sources, Duke Forest. Right: randomly distributed seed sources, Coweeta.

Figure 1 shows the simulation results for scenarios 1 and 2. Solid lines indicate the average relatedness at each distance class for each dispersal scenario, dotted lines the minimum and maximum. Notice that SGS is expected to be very low and flat when the first generation derives from long-distance seed dispersal from outside the study site, regardless of the *average* dispersal distance. SGS is expected to be much more pronounced in cohorts deriving primarily from a few in-plot sources (<5 trees/ha), such as DF generation 2 and C generation 1. Provided there is at least one in-plot source tree, the lower the number of in-plot sources, the stronger the SGS (Figures S1 & S2). SGS is expected to decline in subsequent generations as more seed shadows overlap. Where dispersal distances are long, SGS is weak but is detectable out to longer distances, whereas when dispersal distances are short SGS tends to decline steeply with increasing distances between individuals.

### Spatial genetic structure – analysis of microsatellite data

We examined microsatellite data using the program Genalex [81]. This program, developed specifically for multi-locus multi-allele data, calculates a genetic correlation coefficient *r* for individuals within a series of distance classes, and constructs confidence intervals using a bootstrap approach. Note that *r* is *not* expected to be the same as the mean coefficient of relatedness calculated above. For instance, due to stochasticity in inheritance siblings may not share exactly 50% of their alleles. However, like the coefficient of relatedness, *r* should be high when many individuals in a distance class are related and low when very few individuals are related.

## Results

### Parentage and dispersal

At Duke Forest, 37% of seedlings were estimated by the Bayesian model to have both parents outside the mapped stand when all species were included in the analysis (Table 2). At Coweeta, by contrast, only 7.8% of seedlings had both parents outside the mapped stand, even though the stand was smaller. When species were analyzed separately (ie. assuming no hybridization), the percentage of seedlings with both parents outside the mapped stand increased to 42.9% overall at Duke Forest and 24% overall at Coweeta. For all species except *Q. rubra* at Duke Forest, the percentage of seedlings with both parents in the stand decreased and the percentage with both parents outside the stand increased when we assumed no hybridization; this was particularly pronounced for *Q. velutina* at Coweeta and *Q. falcata* at Duke Forest (Table S2).

**Table 2:** Model results.

	Duke Forest	Coweeta
**u_s_ posterior mean**	6300	92
u_s_ 95% CI	5380–7220	54–130
Expected seed dispersal distance	125 m (95% CI: 115–133 m)	15 m (95% CI: 12–18 m)
**u_p_ posterior mean**	12900	8600
u_p_ 95% CI	11880–13920	7440–9760
Expected pollen dispersal distance	178 m (95% CI: 171–185 m)	146 m (95% CI: 135–155 m)
**Parentage**		
2 in-plot parents	35 seedlings (16%)	41 seedlings (22.9%)
In-plot father, out-of-plot mother	43 seedlings (19.6%)	32 seedlings (17.9%)
In-plot mother, out-of-plot father	60 seedlings (27.4%)	92 seedlings (51.4%)
2 out-of-plot parents	81 seedlings (37%)	14 seedlings (7.8%)

**Figure 2:**
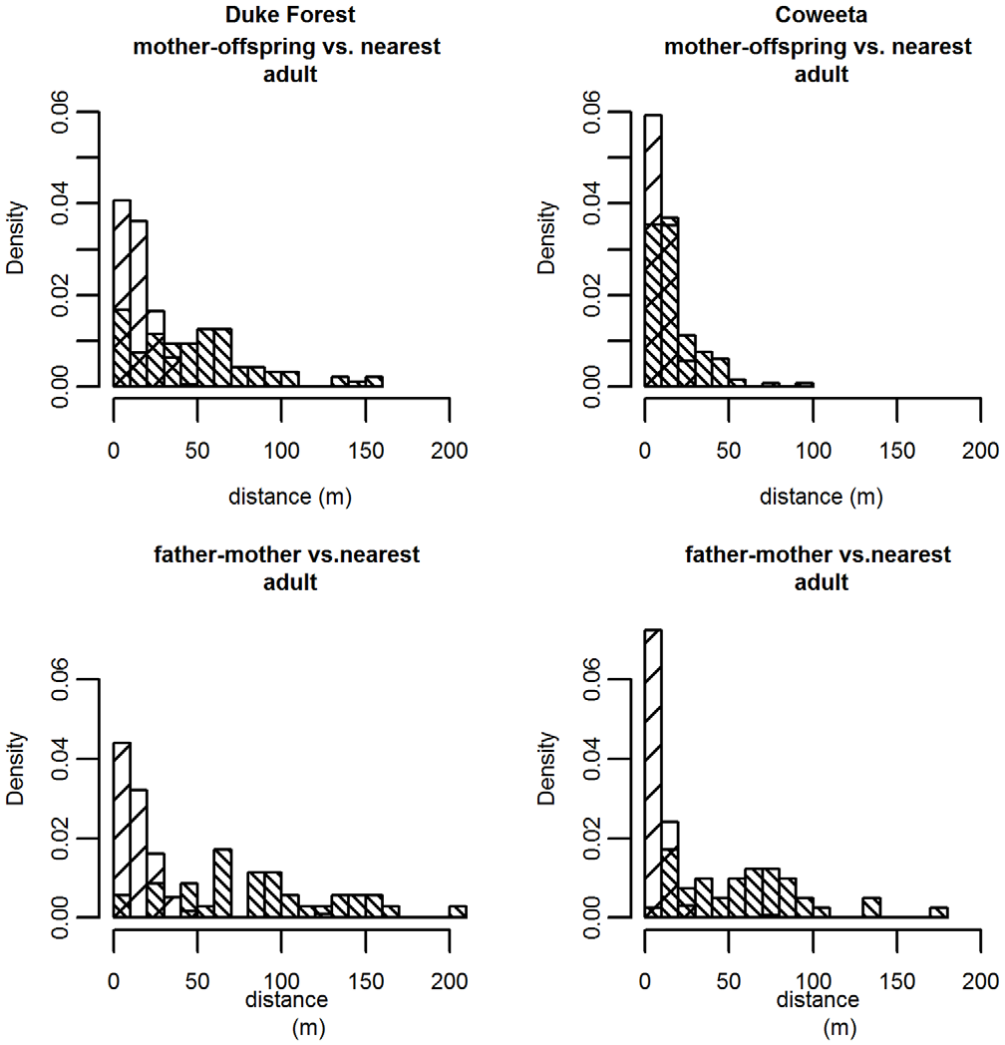
Observed within-stand dispersal distances vs. nearest adult distances. Top – Distances between observed mother-seedling pairs (dark bars) vs. seedling to the nearest adult tree (light). Bottom – Distances between observed mother-father pairs (dark bars) vs. mother tree to nearest neighbor (light bars). Notice that observed dispersal distances are much longer than the nearest-adult-neighbor distance, except for seed dispersal at Coweeta.

The observed distances between seedlings and within-stand mothers when hybridization is assumed (dark hatched bars) are shown for both sites in the top panels of Fig. 2. Distances between within-stand mothers and fathers (dark hatched bars) are shown in the bottom panels of Fig. 2. For comparison, the distances to the nearest adult neighbor (the closest potential seed or pollen donor) are depicted by light hatched bars. Virtually all adults and all seedlings are within 50 m of an adult red oak. Notice that the average mother-offspring and father-mother distances are much greater than the distance to the nearest tree, with the exception of mother-offspring distance at Coweeta.

**Figure 3:**
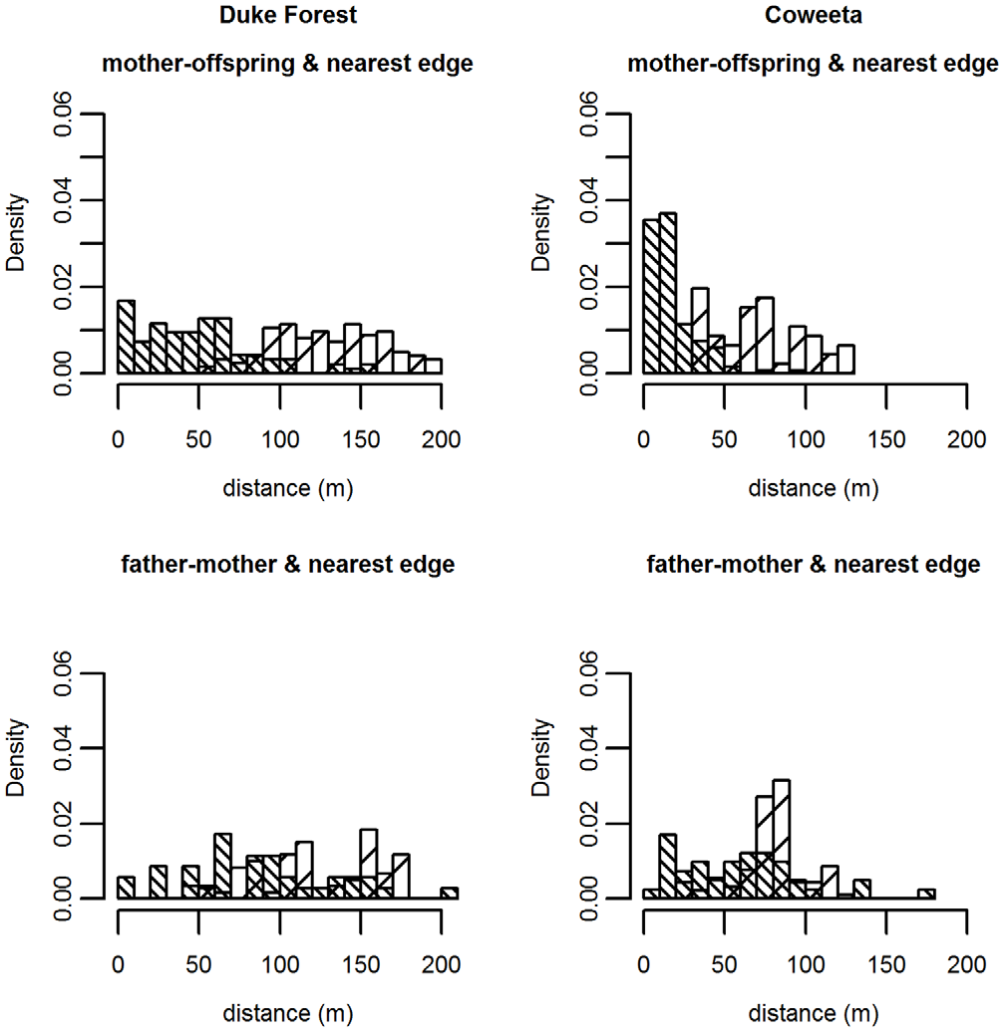
Observed within-stand dispersal distances (dark) and minimum dispersal distances for immigrant seed and pollen (light). Top – Distances between in-plot mother-seedling pairs (dark bars) and between seedlings with an out-of-plot mother and the nearest plot edge (light bars). Bottom – Distances between in-plot mother-father pairs (dark bars) and between mother trees paired with an out-of-plot father and the nearest plot edge (light bars). The mean dispersal distance for both seed and pollen increases when out-of-plot parentage is considered.

At Coweeta, when hybridization is allowed, the posterior mean for the dispersal parameter *u_s_* yields an expected seed dispersal distance of 15 m, which is only slightly larger than previously estimated for gravity-mediated dispersal using seed-trap data from the same sites (*u_s_* = 34.9, expected distance = 9.27 m) [66]. At Duke Forest, however, the estimated *u_s_* corresponds to an expected dispersal distance of 125 m, indicating that most established seedlings are located far beyond the maternal crown (Table 2). Because the dispersal kernel includes dispersal from outside the stand, these expected dispersal distances are longer than the median distances between within-stand mothers and their offspring (10.7 m at Coweeta, 48.5 m at Duke Forest). The distance to the nearest stand edge represents the minimum dispersal distance for immigrant seedlings (Fig. 3, top). If we include these distances, the median observed mother-offspring distance increases to 14.5 m at Coweeta and to 94.9 m at Duke Forest. The true mother-offspring distance for seedlings with out-of-plot mothers is likely to be greater than the distance to the nearest edge. When hybridization is not allowed, dispersal parameter estimates tend to be somewhat smaller, though seed dispersal estimates for Duke Forest are still higher than for Coweeta (Table S2). Model convergence was poor for *Q. velutina* and *Q. coccinea* at Coweeta, most likely due to small sample sizes.

**Figure 4:**
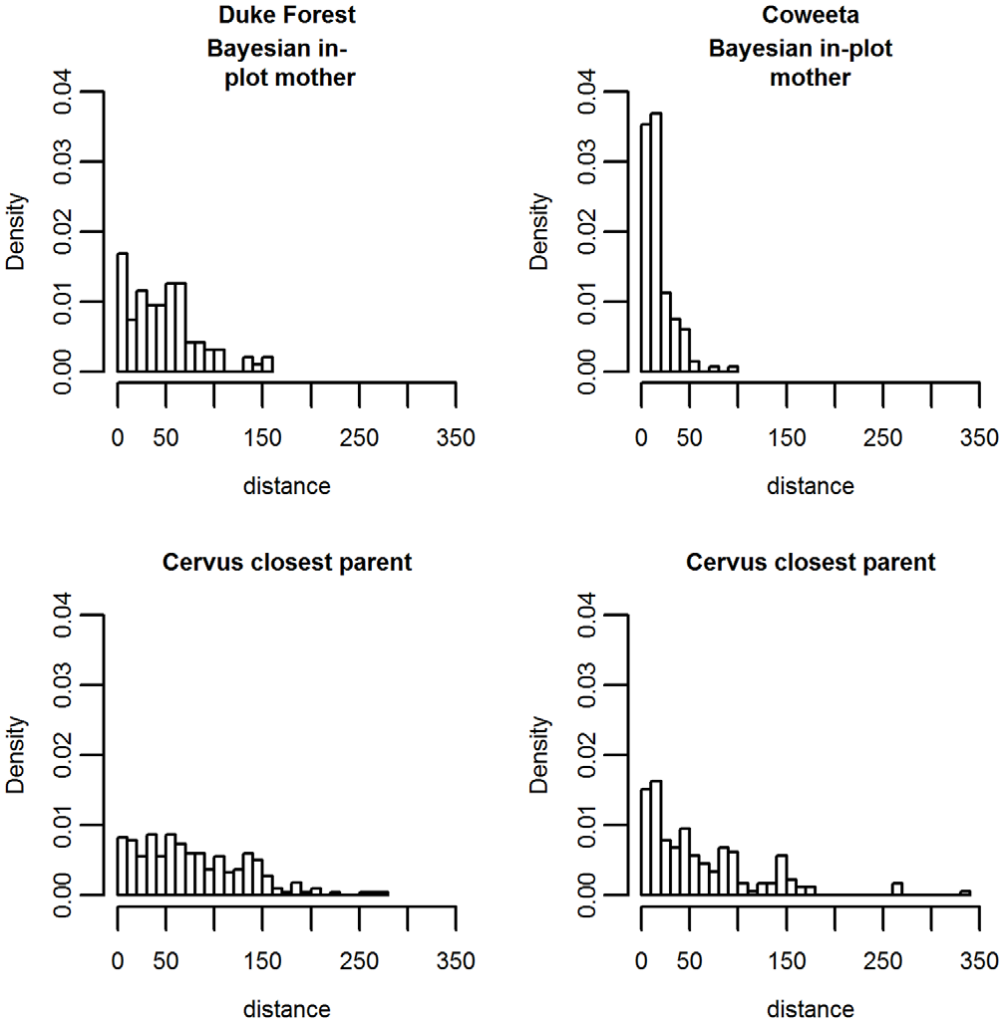
Within-stand mother-offspring distances as estimated by Bayesian model vs. nearest-parent distances as estimated by CERVUS. The Bayesian model rejects many of the parent matches identified by ML approach, due to low seed production, the necessity of assuming high genotyping error, and/or the proximity of a seedling to the stand edge (and potential sources of immigrant seed). However, both models suggest higher seed movement at Duke Forest relative to Coweeta.

Based on allele frequencies and error rates, the expectation using CERVUS was that 58% of seedlings at Duke Forest and 18% of seedlings at Coweeta would be assigned under “strict” criteria (95%), with 74% and 47%, respectively, assigned under “relaxed” criteria (80%), with 26% at Duke Forest and 53% at Coweeta exhibiting lower LOD scores resulting in ambiguous assignments. In reality, matches were much weaker, with only 10% at Duke Forest and 6% at Coweeta meeting the strict match criteria and 64% and 70% remaining unassigned even under the relaxed criteria. Thus, the results of both models reflect the relatively poor genetic match between many seedlings and adult trees within the stand. Our Bayesian model identified 35 seedlings at Duke Forest and 41 seedlings at Coweeta as having 2 in-plot parents. Of these seedlings, the Bayesian model and CERVUS assigned 19 (54.3%) at Duke Forest and 19 (46.3%) at Coweeta the same parent pair; For a further 13 (37.1%) at Duke Forest and 17 (41.5%) at Coweeta, the Bayesian and CERVUS models agreed on one of the in-plot parents but disagreed on the other. For 64 seedlings at Duke Forest and 61 seedlings at Coweeta, the Bayesian model assigned one of the same parents as CERVUS, but the other parent was designated “out of plot”. Overall, the two models agreed on at least one parent for 44% of seedlings at Duke Forest and 54.2% of seedlings at Coweeta. The reason for the disagreements lies in the different assumptions made by the two models. At Duke Forest and Coweeta, respectively, 36.1% and 25.2% of the differing assignments occurred because a parent assigned the highest likelihood by CERVUS had extremely low fecundity (<50 seeds/year); 16.1% and 13% because the parent-offspring pairing would require 3 or more genotyping errors; 5.7% and 25.2% because the pairing would require 1–2 genotyping errors and the seedling was closer to a plot edge than to the proposed parent; and 34.5% and 20.2% because the pairing would require at least one mistyping error of >2 bp. The remaining disagreements, 7.6% at Duke Forest and 16.4% at Coweeta, could not be attributed to a single difference in model assumptions, but involved some combination of ungenotyped loci, genotyping error, fecundity, and distance. The median distance to the nearest CERVUS parent was 42.8 m at Coweeta and 67.5 m at Duke Forest (Figure 4).

Estimates of the pollen dispersal parameter *u_p_* were high for both sites, as expected for a wind-pollinated tree, corresponding to an expected dispersal distance of 146 m at Coweeta and 178 m at Duke Forest (Table 2). A majority of seedlings at both sites are estimated to have fathers outside the mapped study area. As with seed dispersal, expected pollen dispersal distances are longer than the median distances between within-stand mothers and fathers (57 m at Coweeta, 89 m at Duke Forest). Including the distance to the nearest stand edge for mothers pollinated by outside fathers (Fig. 3, bottom) increases the median father-mother distance (to 77.5 m at Coweeta and 105.3 m at Duke Forest). Again, recall that most outside fathers will be further away than the edge of the mapped stand. Again, when hybridization is not allowed, dispersal parameter estimates tend to be somewhat smaller, though pollen dispersal estimates are higher than seed dispersal estimates (Table S2). Model convergence was poor for Q. velutina at Duke Forest and for Q. velutina and Q. coccinea at Coweeta, most likely due to small sample sizes.

### Disperser Density

The recommended uniform/cosine model was favored by AIC. The best-fit model estimate of squirrel density was somewhat higher at Duke Forest than at Coweeta (64.2 vs. 43.7 squirrels/km^2^), but confidence intervals overlapped widely (Table 3).

**Table 3:** DISTANCE results.

Site	Distance function	AIC	Density (squirrels/km^2^)	Upper-Lower CI
**Coweeta**	Uniform/cosine	104.19	**43.7**	**28.6–66.8**
	Half-normal/Hermite	105.06	59.6	31.7–111.0
	Uniform/Polynomial	104.10	**43.7**	**28.6–66.8**
**Duke Forest**	Uniform/cosine	147.4	**64.17**	**31.7–129.9**
	Half-normal/Hermite	149.01	58.2	28.7–117.8
	Uniform/Polynomial	148.66	42.7	1.4–85.1

### Spatial genetic structure

**Figure 5:**
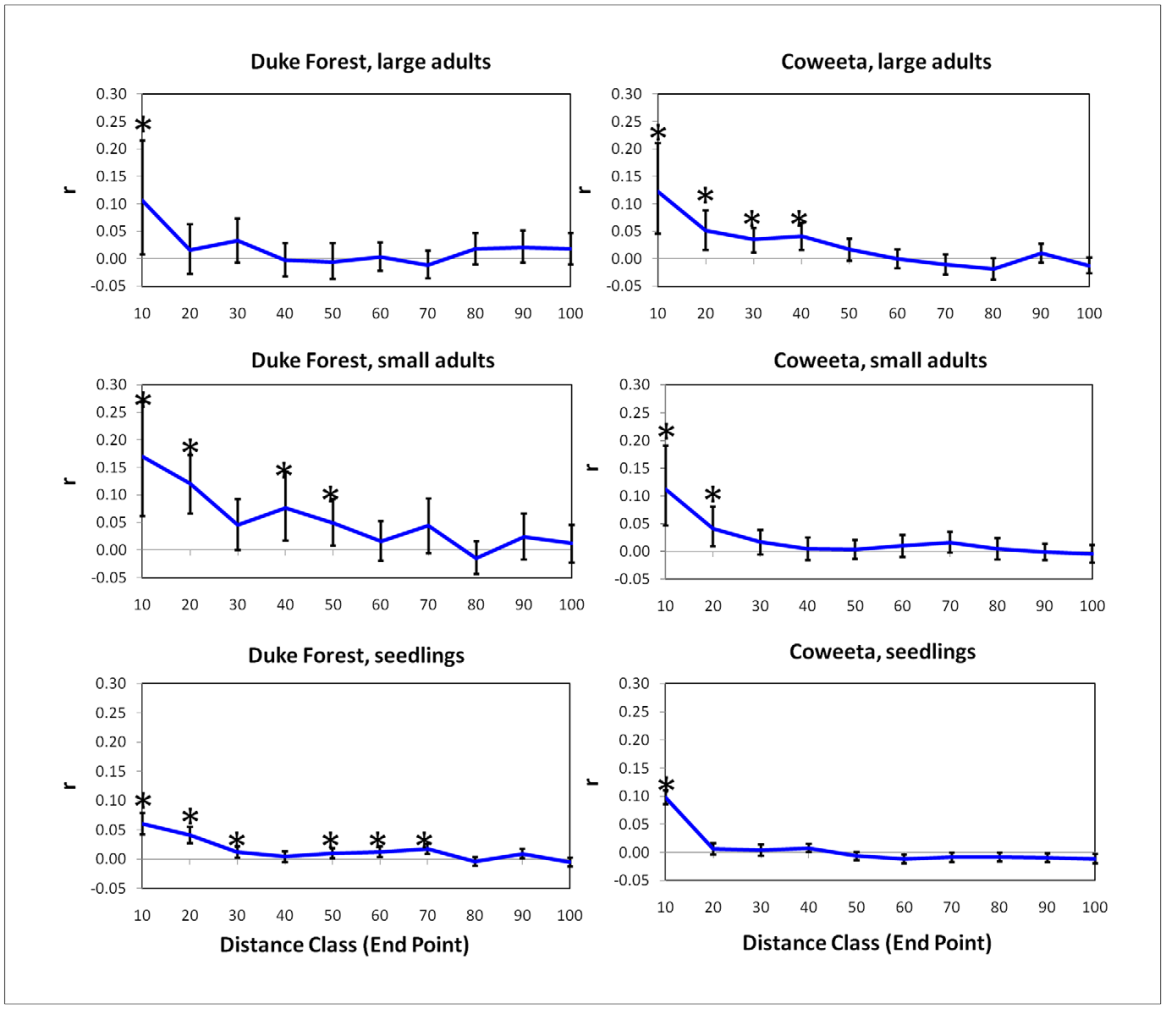
Spatial Genetic Structure results. Correlation in genotype at different distance classes for large adults, small adults, and seedlings. Bars indicate bootstrapped 95% CI. If bars do not overlap zero, this indicates a significant correlation at that scale (indicated with asterixes).

At Duke Forest, the largest 50% of trees exhibit only weak correlation in genotype at the 0–10 m scale, and no correlation at larger distances (Fig. 5A), while the smallest 50% of trees exhibit much stronger SGS (Fig. 5B). Although diameter is a relatively weak proxy for age, this is consistent with hypothesis 3a: that if the first colonists of a site derive from distant sources, SGS should be weak initially and increase over time. At Coweeta, however, this pattern is reversed, with large trees exhibiting significant SGS at a larger spatial scale than small trees (Fig. 5 D&E). This is consistent with hypothesis 3b: that if the oldest trees derive from scattered seed sources inside and outside the plot, and if seed dispersal distances are low, then SGS should be more pronounced in the 1^st^ cohort than the 2^nd^ cohort. Seedlings at both sites exhibited slightly weaker SGS than the small adults (Fig. 5 C&F).

Although some temporal fluctuations in dispersal cannot be ruled out, observed SGS was also consistent with relatively long-distance dispersal (mean>40 m) at Duke Forest and relatively short-distance dispersal (mean<40 m) at Coweeta over the past 90 years (compare Figures 1 and 5). Of the alternate scenarios examined, scenarios 5, 6, and 7 (which include seed sources within the DF study site) would all result in very high SGS in the 1^st^ generation at Duke Forest (Figs. S1,S2). Conversely, scenario 4 (only distant seed sources for C) would result in very low SGS at Coweeta (Fig. S1). We do not observe either of these patterns.

## Discussion

Our results show that the scale of effective seed dispersal can vary substantially between oak populations. At Coweeta, the effective seed dispersal kernel did not differ greatly from the initial gravity-created dispersal kernel estimated from seed trap data (expected distance = 9 m). Although some of the seedlings at this site likely grew from animal-dispersed acorns (see Fig. 3), our results suggest that many originated from seeds that sprouted where they fell, close to the maternal tree. At Duke Forest, by contrast, the effective dispersal kernel is quite flat. This suggests that most of the seedlings at Duke Forest originated from animal-dispersed seeds transported beyond the maternal crown. The median distances to nearest parent, as identified by CERVUS, are larger than the median distances to Bayesian within-plot mother because the Bayesian model tends to assign out-of-plot mothers when a seedling is a poor match to in-plot adults, especially when the seedling is closer to the plot edge than to a potential parent with discrepancies in genotype or low fecundity. However, both models agreed that seed dispersal distances were greater at Duke Forest (Figure 4).

Consistent with previous paternity analyses in oaks [15], [18], [82], [83], LDD of pollen was common at both sites. Indeed, microsatellite data has revealed that long-distance effective pollen dispersal is common in both wind and animal-pollinated plant species [20]. Both sites exhibit high genetic diversity for all age classes (14–35 alleles per locus). LDD of pollen is thought to help maintain the high genetic diversity and low levels of isolation-by-distance observed in many oak species [31], [63], [82], [84].

Unsurprisingly, given our previous analysis suggesting ongoing hybridization [58], the proportion of seedlings matched to within-stand parents tends to decrease when we consider only one oak species at a time per site. Seed and pollen dispersal estimates were somewhat lower when we assumed no hybridization, for two reasons. First, considering only one species at a time tended to decrease the number of seedlings with a good match to a within-stand parent. Second, the density of potential parents is lower, thus decreasing the expected amount of seed and pollen coming from surrounding areas outside the plot. Thus, while the model assuming hybridization might match a *Q. velutina* seedling with a *Q. rubra* tree 250 m away with a single allelic mismatch rather than a *Q. velutina* tree 80 m away with two allelic mismatches or a hypothetical out-of-plot tree 100+ m away, the single-species model must choose between the closer *Q. velutina* and the out-of-plot tree. Because the expected amount of outside seed and pollen has been reduced, the single-species model is more likely to favor the closer conspecific than the multi-species model, unless the genetic match is really bad. However, convergence of dispersal parameter estimates tended to be poor if there were fewer than 20 sampled seedlings or 25 adults of a given species in the dataset.

Transect survey data suggested that squirrel densities might be slightly higher at Duke Forest than at Coweeta but, possibly due to small sample sizes, confidence intervals overlapped widely. Consistent with hypothesis 2, dispersal distances were shorter at the site with highest acorn abundance (Table 1), as estimated based on a previous joint Bayesian growth/fecundity analysis using seed-trap data [4], [85]. At Coweeta, the density of oaks is almost three times higher than at Duke Forest (Table 1), and the average number of red oak acorns produced per hectare per year is 2.3 times higher. This could lead to predator/disperser satiation even in the absence of different disperser densities. Squirrels move seeds much shorter distances in years of high seed production [12]; a similar difference may exist between stands with different levels of acorn production across years. More direct observations of disperser behavior would be necessary to fully test the importance of disperser abundance vs. stand structure.

A few caveats must be mentioned. First, we assumed either free gene flow between co-occurring morphospecies or no gene flow, though there are likely some weak barriers to hybridization [58]. In future analyses it would be desirable to estimate weighting parameters to account for lower inter-specific pollination success [86], but a larger dataset is necessary to obtain good estimates. Second, our plots were larger than those used in many previous dispersal analyses in Fagaceous species [15], [18], [82], [87], but the long dispersal distances detected suggest that larger censused areas (15–30 ha) would be helpful in measuring the tail of the dispersal kernel. Third, we compared only two sites. To date, few genetic dispersal studies have compared more than one site [8], [15], [24], [39], because genotyping is expensive and time-consuming relative to traditional ecological methods; for a given level of effort or funding there is a tradeoff between the number of trees genotyped or the total area censused at one site and the number of sites analyzed. It is to be hoped that reduced genotyping costs in the future will ease this constraint.

As our simulations demonstrate, both dispersal distance and initial conditions can have a strong impact on SGS. At the time the oldest canopy oaks were establishing in the former farmland that became Duke Forest, there were few nearby seed sources. Mixing of multiple distant seed sources is expected to produce low levels of genetic structure in the first generation [34], while matings between the first colonists and local seed dispersal is expected to increase SGS in the 2^nd^ generation [36]. SGS then weakens over time, especially when dispersal is extensive [88]. This is the pattern we observe; alternate scenarios for re-colonization that include local seed sources would all lead to higher levels of SGS in the older trees. At Coweeta, on the other hand, saplings remaining after selective harvests would be expected to serve as local seed sources once mature, leading to high SGS in the first generation [36]; with further dispersal and immigration, SGS should weaken over time. Our observations conform to these expectations. The observed patterns of SGS also suggest that the scale of seed dispersal at Duke Forest has long been larger than at Coweeta. If average dispersal distances >40 m had occurred at Coweeta, we would expect to see weak SGS extending to longer distances in the first cohort, and virtually no SGS in later cohorts, due to overlapping seed shadows. Similarly, if the second cohort at Duke Forest had derived from highly restricted seed dispersal, we would expect to see stronger correlation in genotypes at short distances. If higher acorn production at a site does lead to shorter dispersal distances, this could partly explain the consistency over time; because Coweeta was not completely cleared, oak densities have probably been higher for many years. In a study of *Fagus sylvatica* and *F. crenata*, Oddou-Muratorio et al. [8] also found that gene flow estimates calculated from parentage/dispersal analyses based on young seedlings and from the SGS of adults were on a similar scale.

It should be noted that our simulations used average coefficient of relatedness, not *r*, as a measure of SGS. This was done to avoid having to simulate both parentage and a multi-locus genotype for each individual, which would have slowed computation excessively. Therefore, we have only made a *qualitative* comparison between the model predictions and the observed patterns of SGS. SGS within a cohort tends to weaken over time due to self-thinning [89], [90]. Our simulations controlled for this by generating patterns of relatedness for adults (based on actual adult locations) in generations 1 and 2. Our sites were located in long-established research areas and better historical records were available than for many forests, but the exact history of oak regeneration is unknown. When the location of source populations and ages of sampled trees are known (e.g. [35], [36]), this data should be included in the analysis.

Most previous parentage and dispersal studies in Fagaceous trees have reported relatively restricted seed dispersal distances compared to pollen dispersal distances. For instance, average within-stand mother-offspring distances (assuming the closest parent was the mother) compared to father-mother distances were 26 m versus 76.9 m for *Q. macrocarpa* saplings [18], and 16.8 m versus 69.2 m for *Q. salicinia* seedlings [17]. Studies using the full-probability seedling neighborhood model [47] reveal similar patterns: average within-stand dispersal distances of 10.4–64.2 m for seed vs. 28.1–79 m for pollen in 3 *Fagus sylvatica* stands and one *F. crenata* stand [8], and average within-neighborhood dispersal distances of 4.4–7.3 m for seed vs. 17.3–29 m for pollen in 2 mixed-species *Q. robur*/*Q.petraea* stands [15]. In this study, the average within-stand dispersal at Coweeta was typical for Fagaceae (10.7 m for seed, 57 m for pollen), while the within-stand dispersal at Duke Forest was at the upper end of the range of previous measurements (48.5 m for seed, 89 m for pollen). Of course, dispersal estimates tend to increase when immigration is considered. Previous studies have reported seed immigration rates of 4.5–15% and pollen immigration rates of 52.1–71% for *Quercus* [15], [17], [18] and seed immigration rates of 0.7–36% and pollen immigration rates of 40–72% for *Fagus* [8]. Our estimates for pollen immigration are within this previously observed range (58.9% at Coweeta, 64.4% at Duke Forests), but our estimates of seed immigration at both sites are high (25.7% at Coweeta, 56.5% at Duke Forest), in part because our model does not assume that the closest parent or the sole within-stand parent is the mother – seedlings with a father but not a mother within the stand can be counted as “seed immigrants”. Chybicki and Burczyk [15] used a 2-part kernel incorporating both short-range and long-range dispersal to estimate average dispersal distances that include immigration; their estimates of seed dispersal in *Q.robur/Q.petraea* (8.8–15.6 m) were short relative to our Duke Forest estimate, but their estimates of mean pollen dispersal were extremely high (297–463 m).

The highest previous seed dispersal estimates for *Quercus* or *Fagus* were derived from older seedlings or saplings [8], [18], so it is possible that density- or distance-dependent mortality [91], [92] could inflate estimates of seed dispersal. In such a case, higher survival of seedlings far from parents would increase the apparent dispersal distance over time. However, the seedling population at Duke Forest is younger than that at Coweeta. At Duke Forest, 10% of samples seedlings emerged between 2007 and 2008; based on bud scar number, 60% recruited 2004–2006, 28.7% recruited 2001–2003, and only 1.7% recruited prior to 2001. At Coweeta, only 0.5% of seedling were observed to recruit between 2007 and 2008; based on bud scars, 25.6% recruited 2004–2006, 61.2% recruited 2001–2003, while 4.4% recruited prior to 2001. Note that the ages of older seedlings are only approximate – bud scars may be lost if an individual dies back and resprouts. Many of the 2001–2003 cohort at Coweeta are likely the result of a large mast event in 2000. While there were insufficient “new” seedlings to do a separate analysis, the relative youth of Duke Forest seedlings, together with low mortality rates for both new and established seedlings at this site (Table 1) and the proximity of most seedlings to adult oaks at both sites (Figure 2), suggests that distance- or density-dependent mortality is not the primary cause of the between-site difference in dispersal estimates. Moreover, most seedlings were less than 20 m from an adult and virtually all were within 50 m of an adult tree, yet annual survival was high even when seedling densities were high, as at Coweeta. At Duke Forest and Coweeta, respectively, first year survival was 85% and 67%, while established seedling annual survival was 96% and 98%. Nevertheless, it would be instructive to compare our results to data from new seedlings following a mast year; for species with low seedling survival, sampling new germinants is essential.

How might longer dispersal distances in some oak populations, such as observed using genetic data at Duke Forest, affect our understanding of their migration ability? Clark et al. [6] showed that when migration rate is defined by the position of the furthest-forward individual and dispersal follows a fat-tailed 2D-t kernel, then the asymptotic wave speed is approximately equal to:
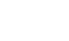
where *T* is the generation time, *u* is the dispersal parameter, and *R_0_* is the expected number of offspring at birth (that is, expected lifetime reproduction given pre-reproductive mortality). The asymptotic spread rate is based on spread by “jumps” from the furthest forward individual; when the source population is large, as is often the case early in the migration process, spread rates are faster. We can calculate a ratio between the minimum spread rates for two populations with different dispersal parameters:
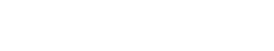
From this, we can see that the asymptotic spread rate based on the dispersal estimate for Coweeta (*u_s_* = 92) is 1.6 times higher than that based on seed trap estimates (*u_s_* = 34.9). The asymptotic spread rate based on the Duke Forest estimate (*u_s_* = 6,300) is 8.3 times higher than for Coweeta.

However, these increased dispersal estimates (relative to seed trap estimates) do not necessarily mean that oak species will be able to keep pace with climate change. First, it is not known what proportion of oak populations exhibit restricted (Coweeta-like) vs. extensive (Duke Forest-like) seed dispersal. Second, even using the unexpectedly high seed dispersal estimates from Duke Forest, asymptotic spread rates are relatively slow. A recent study estimated that under the A1B emission scenario average temperatures would shift at an average rate of 420 m/yr; in temperate broadleaf forests the rate of change averaged 350 m/yr, but in some areas exceeded 1,000 m/yr [93]. If we assume 99.9% mortality between seed dispersal and adulthood, a 20-year generation time, an average fecundity of 800 seeds/yr once mature, and an 80-year reproductive life [6], then the asymptotic spread rate for the Coweeta population would be 4.8 m/yr and for the Duke Forest population 39.8 m/yr. Even if one made the optimistic assumption that the generation time is 15 years, trees produce 2000 seeds/yr, and reproduce for 85 years, the Duke Forest spread rate would be only 86.5 m/yr. These rates would likely be sufficient for altitudinal range shifts, but are slow relative to predicted latitudinal shifts in climate [93]. In addition, Clark et al. [6] showed that, if variability in R_0_ due to stochastic mortality is taken into account, migration is up to 2 orders of magnitude slower than predicted based on average R_0_.

Because of the time and cost involved, most gene-marker-based dispersal studies in trees are based on a single population [94]. Although a clear picture of geographic variation cannot be derived from just two sites, the differences between sites in our study and some previous analyses [15], [39] illustrate the potential for wide site-to-site variation in forest tree dispersal ability. Gene flow needs to be considered in a broader context, especially in widespread species, in order to better understand population dynamics and the potential for population spread in woody plants. However, results to date suggest that even under favorable conditions migration rates in nut-bearing trees are likely to lag contemporary climate change.

## Supporting Information

Text S1 **Dispersal Model Implementation.**
(DOC)

Text S2 **Spatial Genetic Structure Simulations.**
(DOC)

Figure S1 **Distant-source simulations.** Left column – distant sources, Duke Forest. Middle column – distant sources plus 3 in-plot source trees, Duke Forest. Right column – distant sources, Coweeta. Top row – Blue dots indicate simulated original source trees, red large adult trees (“Generation 1”), green small adult trees (“Generation 2”). Middle and bottom rows –average coefficient of relatedness when generations 1 and 2 for *u_s_* = 20 (blue), 100 (red), 800 (purple), 3500 (green), or 7000 (black).
(TIF)

Figure S2 **Scattered-source simulations.** Left column – 0.5 source trees/ha, Coweeta. Middle column –1.5 source trees/ha, Coweeta. Right column – 0.5 source trees/ha, Duke Forest. Top row – Blue dots indicate simulated original source trees, red large adult trees (“Generation 1”), green small adult trees (“Generation 2”). Middle and bottom rows –average coefficient of relatedness for generations 1 and 2 when *u_s_* = 20 (blue), 100 (red), 800 (purple), 3500 (green), or 7000 (black).
(TIF)

Table S1 **Disperser transect survey data.** Shown: date of survey, total length of transects, observation time, total number of squirrels, squirrels per hour of observation.
(DOC)

Table S2 **Results of single-species dispersal analyses.** The expected dispersal distances, the range of expected dispersal distances corresponding to the 95% CI of the dispersal parameter, and the number of seedlings with a given number of parents within the mapped stand are shown for single-species (“separate”) and multi-species (“joint”) analyses. An asterix denotes poor model convergence due to low sample size.
(DOC)
