# Supplementary material for: Between-Site Differences in the Scale of Dispersal and Gene Flow in Red Oak
Source: PLoS One. 2012 May 1;7(5):e36492. doi: 10.1371/journal.pone.0036492 (PMC3341347; doi:10.1371/journal.pone.0036492)
Supplement: Text S1 — Dispersal Model Implementation. (DOC) [file pone.0036492.s001.doc]

Dispersal Model Implementation

The dispersal kernel Other dispersal kernels other than the 2D-t could be used in this modeling framework; we selected the 2D-t to allow comparison with previous, non-molecular analyses at these sites , because seed and pollen dispersal in trees are usually well-described by such fat-tailed kernels , and because distributions with a larger number of parameters would be difficult to fit with the amount of data available. Seed production and probability of maturity were estimated based on a separate study; both are positively related to dbh . Little individual-level data on male vs. female reproductive allocation exists for trees . Therefore, pollen production was assumed to be proportional to seed production, as it is likely that large trees producing many seeds also produce large amounts of pollen.

Immigrant seed and pollen This model includes dispersal of seed and pollen from outside the mapped area. Because both stands are part of a continuous forest, we can substitute area at a given distance for the specific position and fecundity of out-of-plot trees via numerical integration, using the same seed and pollen dispersal kernels to calculate the expected amount of pollen or seed reaching a given point originating from inside vs. outside the mapped stand. This procedure is described mathematically and graphically in the Moran and Clark online supplement.

Genotyping error The probability that parents with observed genotypes *G_i_^o^* and *G_i’_^o^* could produce an offspring with observed genotype *G_k_^o^* depends on Mendelian inheritance probabilities and on the probability of genotyping error. If an individual is not genotyped at a locus, either due to PCR failure or because the tree is a hypothetical out-of-plot parent, the probability that the true genotype is (a_1_,a_2_) = freq(a_1_)freq(a_2_), where freq(a_x_) is the frequency of allele a_x_ in the population.

Priors Priors for dispersal parameters were constructed based on data from the literature: u_s_ ~ N(253,1000), truncated at 10 and 10,000, while u_p_ ~ N(2000,1500), truncated at 10 and 15,000. The truncation points reflect the fact that *u*’s must be positive, and that mean acorn and pollen dispersal distances are likely to be > 5 m but less than < 160 m and 200 m, respectively (though extreme dispersal events – the fat tail of the distribution – could extend much further). Justification for these prior choice, literature citations, and a discussion of the consequences of prior choice can also be found in the Moran and Clark online supplement. Note that, due to the difference in prior means, the situation in which *i* is the mother of *k* and *i*' the father will be considered somewhat more likely than the reverse when *d_ik_* < *d_i’k_* . However, because the priors overlap broadly, the closest parent is *not* always assumed to be the mother, and the posteriors for the dispersal parameters reflect this uncertainty in maternity vs. paternity.

Model output. Output includes posterior means and credible intervals for the dispersal parameters and seedling parentage. Parentage posteriors can be expressed as 2-dimensional multinomials nadult+1 x nadult+1 in size (to accommodate hypothetical out-of-plot parents), with a probability associated with each mother-father pair. The best estimate of the pedigree for each seedling is the parent pair with the highest posterior probability. If the true parents are located in the mapped stand, the model will nearly always identify them. Simulations indicate that for a range of seed and pollen dispersal parameters, true parents are identified 97% of the time . When the correct parent pair is identified, 88% of the time the estimated mother = true mother and estimated father = true father, while 12% of the time estimated mother = true father and vice-versa. Identification of the dispersal parameters is also highly accurate, though the proportion of the time that the true parameter falls within the 95% CI declines as plot size grows smaller or dispersal parameters grow extremely large.

**References**

1. Clark JS, Silman M, Kern R, Macklin E, H. R. Lambers J (1999) Seed dispersal near and far: patterns across temperate and tropical forests. Ecology 80: 1475-1494.

2. Clark JS, LaDeau S, Ibanez I (2004) Fecundity of trees and the colonization competition hypothesis. Ecological Monographs 74: 415-442.

3. Streiff R, Labbe T, Bacilieri R, Steinkellner H, Glossl J, et al. (1998) Within-population genetic structure in *Quercus robur* L. and *Quercus petraea* (Matt.) Liebl. assessed with isozymes and microsatellites. Molecular Ecology 7: 317-328.

4. Goto S, Shimatani K, Yoshimaru H, Takahashi Y (2006) Fat-tailed gene flow in the dioecious canopy tree species *Fraxinus mandshurica* var. *japonica* revealed by microsatellites. Molecular Ecology 15: 2985-2996.

5. Hardesty BD, Hubbell SP, Bermingham E (2006) Genetic evidence of frequent long-distance recruitment in a vertebrate-dispersed tree. Ecology Letters 9: 516-525.

6. Clark JS, Bell D, Chu C, Courbaud B, Dietze M, et al. (2010) High dimensional coexistence based on individual variation: a synthesis of evidence. Ecological Monographs 80: 569-608.

7. LaDeau S, Clark JS (2006) Pollen production by *Pinus taeda* growing in elevated atmospheric CO2. Functional Ecology 20: 541-547.

8. LaDeau S, Clark JS (2001) Rising CO_2_ levels and the fecundity of forest trees. Science 292: 95-98.

9. Moran EV, Clark JS (2011) Estimating seed and pollen movement in a monoecious plant: a hierarchical Bayesian approach integrating genetic and ecological data. Molecular Ecology 20: 1248-1262.
