# Supplementary material for: Between-Site Differences in the Scale of Dispersal and Gene Flow in Red Oak
Source: PLoS One. 2012 May 1;7(5):e36492. doi: 10.1371/journal.pone.0036492 (PMC3341347; doi:10.1371/journal.pone.0036492)
Supplement: Text S2 — Spatial Genetic Structure Simulations. (DOC) [file pone.0036492.s002.doc]

Spatial Genetic Structure Simulations

We implemented simulations in R. Simulated landscapes included an inner “study stand” set at the center of a 1200 m x 1200 m landscape. First-generation trees were assumed to have the same locations as the largest 50% of trees at each site, second-generation trees the same locations as the smallest 50% of trees, and third-generation individuals the same location as sampled seedlings. The probability that any potential parent *i* is the mother of a particular offspring individual *k* is equal to the proportion of the seed reaching site *k* expected to come from tree *i* relative to the amount of seed coming from all potential parents. Likewise, the probability that a potential parent *i'* is the father is equal to the proportion of pollen reaching mother tree *i* that is expected to come from tree *i'*. By making these assumptions, we were able to a) control for the effect on SGS of tree distribution and mortality from seed to census-age (distances between individuals in each cohort are the same as in the real dataset) and b) reduce computation time (as we need only calculate the probability that a limited number of individuals had a particular set of parents, not the probability that a seed from a given tree reaches *any* spot in the plot and survives to census-age).

Dispersal of seed and pollen was simulated in a manner consistent with the dispersal and parentage model (further discussed in Text S1). Pollen was assumed to follow a 2D-t kernel with *u_p_* = 9000; this gives an expected dispersal distance of 149 m, though the fat-tailed shape of this kernel means that many pollen grains will disperse further. Seed dispersal followed a 2D-t kernel with *u_s_* = 20, 100, 800, 3500, or 7000, corresponding to expected seed dispersal distances of 7 m, 16 m, 44 m, 93 m, and 131 m. Average yearly seed production at both sites roughly follows zero-inflated lognormal distributions, with 30-60% of trees > 10 cm DBH not producing any seed and highly skewed seed production among those trees that do reproduce . In these simulations, we assumed that 30% of trees were non-reproductive, while for reproductive trees average annual seed production (*f_i_*) was drawn from lnN(6.69,0.47), corresponding to an average fecundity of 804 seeds/year – similar to the Coweeta site. Pollen production (*c_i_*) was assumed to be proportional to seed production. Where *d_ik_* is the distance between *k* and putative mother *i*, and *d_i’i_* is the distance between putative parents *i'* and *i*:

$$p(i=mother(k))=\frac{f_{i}p\left(d_{ik}\left|u_{s}\right.\right)}{\sum_{I} f_{i}p\left(d_{ik}\left|u_{s}\right.\right)}$$

$$p(i'=father(k)\left|i=mother(k))=\right.\frac{c_{i'}p\left(d_{i'i}\left|u_{p}\right.\right)}{\sum_{I} c_{i'}p\left(d_{i'i}\left|u_{p}\right.\right)}$$

In each of 100 repeat simulations, mothers and fathers for each individual were drawn from the resulting multinomial distributions. We then determined relatedness between all individuals in each cohort, and calculated the average coefficient of relatedness at each distance class. We assumed that the original source trees are unrelated (coefficient of relatedness for any pair = 0). If individuals have unrelated parents, then for full sibs the coefficient of relatedness is 0.5, for half-sibs 0.25, and for unrelated individuals 0. The coefficient of relatedness between parent and offspring is 0.5. For the second and third generations, the coefficient of relatedness between individuals *i* and *j* is equal to $R_{ij}\left(1+\left(\frac{mean(F_{i},F_{j})}{2}\right)\right)$, where *R_ij_* is 0.5 if *i* and *j* are full sibs and 0.25 if they are half-sibs, *F_i_* is the relatedness of the parents of *i* and *F_j_* is the relatedness of the parents of *j*. For example, if *i* and *j* are full sibs, and their parents were also full sibs, then their coefficient of relatedness is 0.625.

We examined several sets of initial conditions for each site. In the **Duke Forest** **(DF)** **simulations**, source trees were located:

1. Outside the mapped stand in three 300m x 300m patches (density = 5/ha) in upper right, lower right, and lower left corners (**scenario 1,** Figure S3 top left). This situation is similar to what would have occurred if there were no adult oaks in the plot as regeneration began in the 1920’s. Mature oaks are known to exist to the south and to the north-east of the mapped stand, whereas the area to the north-west is at an earlier successional stage.
2. In 3 out-of-plot source populations, plus three in-plot trees (**scenario 5,** Figure S3 top middle). This situation is similar to what would have occurred if some adult oaks had been retained for shade or in selectively cut woodlots.
3. Source trees sparsely scattered across the landscape (**scenario 6,** Figure S4 top right), 0.5 trees/ha. Such a situation could have occurred if some adult oaks were retained within the farmed landscape, but heavy use prevented the buildup of high population densities.
4. Scattered at a moderate density across the landscape (**scenario 7**), 1.5 trees/ha. This situation is less likely, given what is known about the history of the site.

In the **Coweeta (C) simulations**, source trees were:

1. Scattered at a moderate density across the landscape (**scenario 2,** Figure S4 top middle), 1.5 trees/ha. Such a situation is likely given the history of the site, as saplings retained after selective harvests matured and began to produce seed.
2. Sparsely scattered across the landscape (**scenario 3,** Figure S4 top left), 0.5 trees/ha. This scenario is also possible, depending on how heavily oaks were harvested.
3. Located outside the study stand in three 300m x 300m patches (density = 5/ha) in upper right, lower right, and lower left corners (**scenario 4,** Figure S3 top right). This situation is unlikely to have occurred at this site.

Results from some of these simulations are shown in Figure 1 and supplement Figures S3 and S4. Dotted lines indicate the maximum and minimum average coefficients of relatedness, dark lines the mean over all 100 simulations. From these simulations, we can make the following general observations:

1. When seed sources are located outside the plot, SGS is uniformly low in the first generation regardless of the mean dispersal distance (Figure S3, columns 1 and 2).
2. When seed sources are scattered across the entire landscape, SGS tends to be stronger in the first generation at lower seed source densities (Figure S4), most likely because a few in-plot trees are the parents of most of the individuals in this generation. Results are not shown for scenario 7, but SGS was weaker compared to scenario 6 to a degree similar to that shown for scenarios 2 and 3. A similar effect can be seen when both distant sources and a few in-plot sources exist (Figure S3, column 2).
3. When there are multiple seed sources within the plot, short dispersal distances tend to create moderately-strong SGS at short distances, declining quickly with distance. Longer dispersal distances create a flatter pattern of SGS which, while weaker, may be detectible to longer distances.
4. If SGS is high in one generation, it tends to decrease in the next, as more seed shadows overlap. On the other hand, if SGS is very low in the first generation, it tends to increase in the next.
5. Similar patterns of SGS can be obtained from different combinations of dispersal distance and stand structure. For instance, similar patterns of moderately high SGS, declining slowly with distance, can be created either by sparsely scattered seed sources and *u_s_* = 7000 or by more densely scattered seed sources and u_s_ = 800 (Figure S4, row 2).

References

1. Moran EV, Clark JS (in press) Variation in reproductive success in forest trees: its causes and consequences. Ecology.
