# Supplementary figures and images for: Between-Site Differences in the Scale of Dispersal and Gene Flow in Red Oak
Source: PLoS One. 2012 May 1;7(5):e36492. doi: 10.1371/journal.pone.0036492 (PMC3341347; doi:10.1371/journal.pone.0036492)

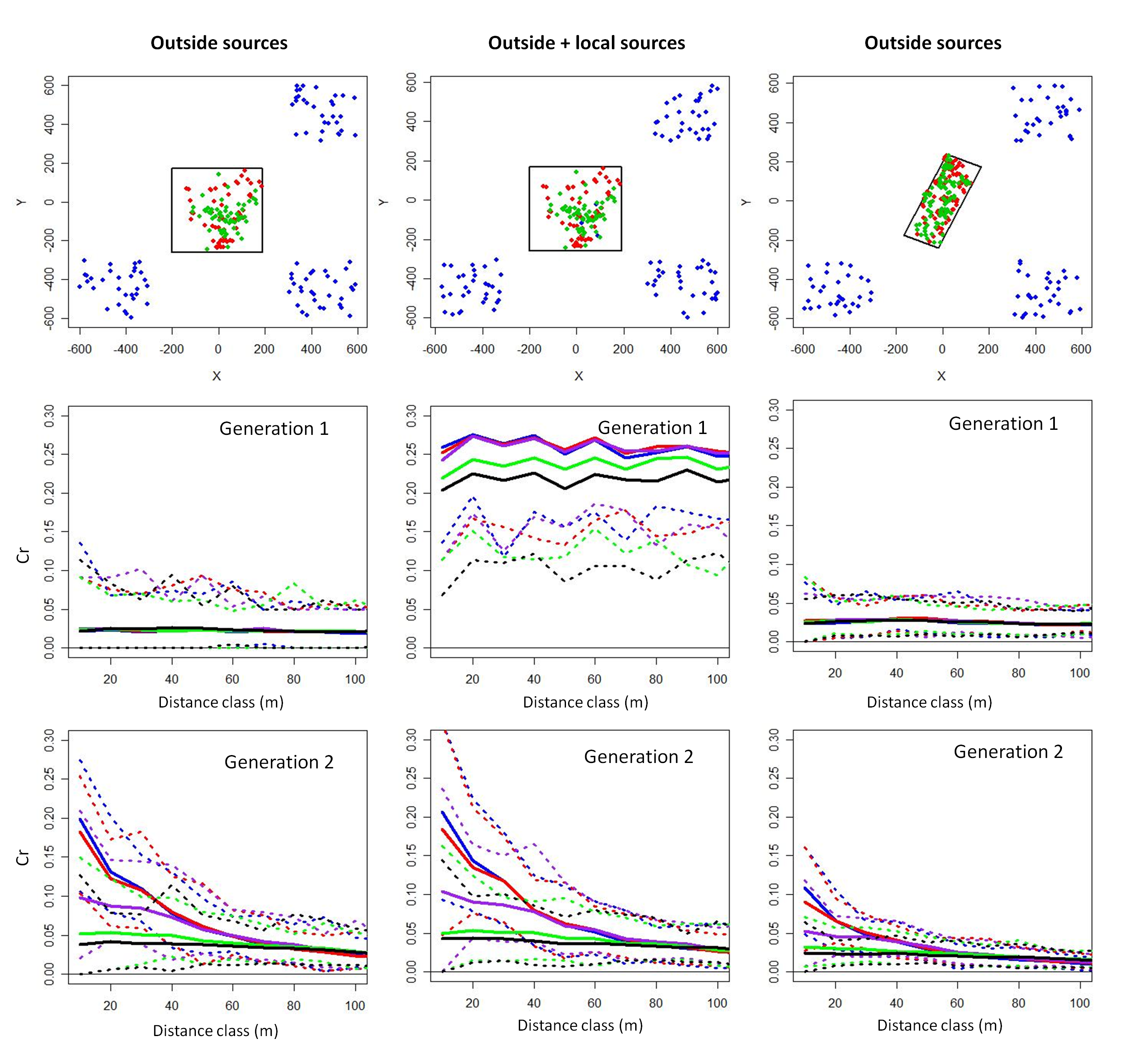

Supplement: Figure S1 — Distant-source simulations. Left column – distant sources, Duke Forest. Middle column – distant sources plus 3 in-plot source trees, Duke Forest. Right column – distant sources, Coweeta. Top row – Blue dots indicate simulated original source trees, red large adult trees (“Generation 1”), green small adult trees (“Generation 2”). Middle and bottom rows –average coefficient of relatedness when generations 1 and 2 for us = 20 (blue), 100 (red), 800 (purple), 3500 (green), or 7000 (black). (TIF) [file pone.0036492.s003.tif]

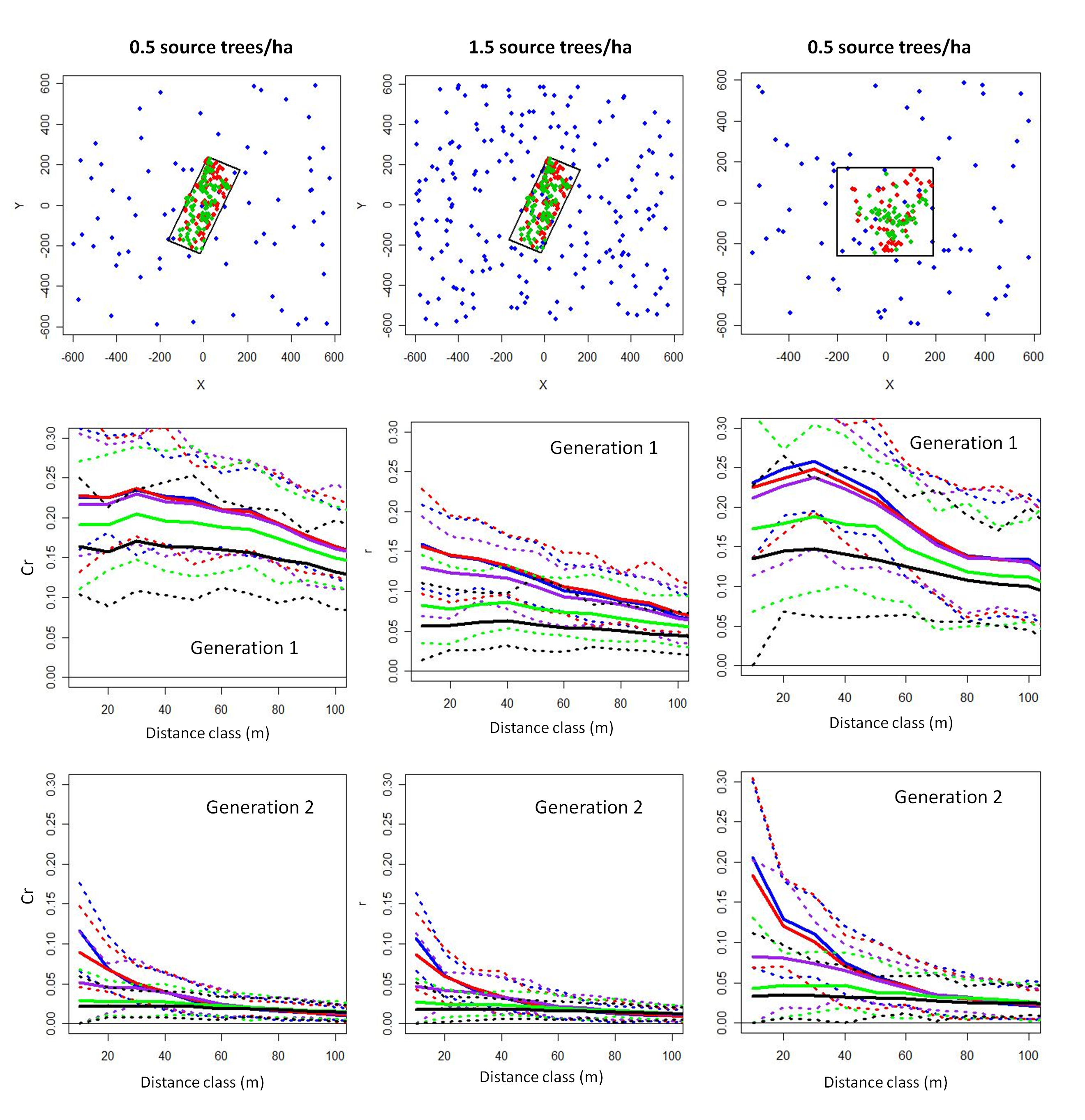

Supplement: Figure S2 — Scattered-source simulations. Left column – 0.5 source trees/ha, Coweeta. Middle column –1.5 source trees/ha, Coweeta. Right column – 0.5 source trees/ha, Duke Forest. Top row – Blue dots indicate simulated original source trees, red large adult trees (“Generation 1”), green small adult trees (“Generation 2”). Middle and bottom rows –average coefficient of relatedness for generations 1 and 2 when us = 20 (blue), 100 (red), 800 (purple), 3500 (green), or 7000 (black). (TIF) [file pone.0036492.s004.tif]
