# Supplementary material for: Between-Site Differences in the Scale of Dispersal and Gene Flow in Red Oak
Source: PLoS One. 2012 May 1;7(5):e36492. doi: 10.1371/journal.pone.0036492 (PMC3341347; doi:10.1371/journal.pone.0036492)
Supplement: Table S1 — Disperser transect survey data. Shown: date of survey, total length of transects, observation time, total number of squirrels, squirrels per hour of observation. (DOC) [file pone.0036492.s005.doc]

Table S1: Disperser transect survey data. Shown: date of survey, total length of transects, observation time, total number of squirrels, squirrels per hour of observation

|  | Date | Time of day | Distance (m) | Total obs time (min) | #squirrels | Squirrels per hour |
| --- | --- | --- | --- | --- | --- | --- |
| Coweeta | 11/06/09 | 7:19 – 9:13 | 541 | 82 | 4 | 2.93 |
|  | 11/06/09 | 14:51-16:11 | 541 | 61 | 3 | 2.95 |
|  | 11/07/09 | 7:15-8:36 | 541 | 61 | 2 | 1.97 |
|  | 11/07/09 | 9:06-10:14 | 541 | 54 | 3 | 3.33 |
|  | 11/07/09 | 15:00-16:19 | 541 | 61 | 2 | 1.97 |
|  | **Mean** |  |  |  |  | **2.63** |
| Duke Forest | *(pilot) 10/21/09* | *8:02-10:01* | *750* | *77* | *7* | *5.45* |
|  | 12/01/09 | 7:37-9:08 | 740 | 67 | 2 | 1.79 |
|  | 12/01/09 | 9:53-11:16 | 740 | 66 | 3 | 2.73 |
|  | 12/01/09 | 15:13-16:31 | 740 | 65 | 2 | 1.85 |
|  | 12/03/09 | 7:38-8:54 | 740 | 66 | 1 | 0.91 |
|  | 12/03/09 | 14:57-16:11 | 740 | 64 | 4 | 3.75 |
|  | **mean**  ***(without pilot)*** |  |  |  |  | **2.75**  ***(2.2)*** |
