# Supplementary material for: Between-Site Differences in the Scale of Dispersal and Gene Flow in Red Oak
Source: PLoS One. 2012 May 1;7(5):e36492. doi: 10.1371/journal.pone.0036492 (PMC3341347; doi:10.1371/journal.pone.0036492)
Supplement: Table S2 — Results of single-species dispersal analyses. The expected dispersal distances, the range of expected dispersal distances corresponding to the 95% CI of the dispersal parameter, and the number of seedlings with a given number of parents within the mapped stand are shown for single-species (“separate”) and multi-species (“joint”) analyses. An asterix denotes poor model convergence due to low sample size. (DOC) [file pone.0036492.s006.doc]

Table S2: Results of single-species dispersal analyses.

|  | **Duke Forest** | | **Coweeta** | |
| --- | --- | --- | --- | --- |
|  | **Separate** | **Joint** | **Separate** | **Joint** |
|  | **Expected seed dispersal distance** | | | |
| All species | - | 125 m | - | 15 m |
| *Q. rubra* | 97 m | - | 9 m | - |
| *Q. velutina* | 103 m | - | 59 m* | - |
| *Q. coccinea/falcata* | 101 m | - | 42 m* | - |
|  | **95% CI expected seed dispersal distance** | | | |
| All species | - | 115 – 133 m | - | 12 – 18 m |
| *Q. rubra* | 82 – 109 m | - | 7 – 12 m | - |
| *Q. velutina* | 85 – 120 m | - | 19 - 80 m* | - |
| *Q. coccinea/falcata* | 79 - 119 m | - | 19 – 61 m* | - |
|  | **Expected pollen dispersal distance** | | | |
| All species | - | 178.3 m | - | 145.6 m |
| *Q. rubra* | 157 m | - | 78.5 m | - |
| *Q. velutina* | 164.7 m* | - | 71.9 m* | - |
| *Q. coccinea/falcata* | 114.3 m | - | 43 m * | - |
|  | **95% CI expected pollen dispersal distance** | | | |
| All species | - | 171-185 m | - | 135 – 147 m |
| *Q. rubra* | 151 – 165 m | - | 67 – 89 m | - |
| *Q. velutina* | 157 – 172 m* | - | 45-87 m* | - |
| *Q. coccinea/falcata* | 95 – 139 m | - | 33 – 83 m* | - |
|  | **Both parents within stand** | | | |
| All species | 41 (18.7%) | 35 (16%) | 28 (15.6%) | 41 (22.9%) |
| *Q. rubra* | 24 (25%) | 17 (17.7%) | 26 (16.4%) | 37 (23.3%) |
| *Q. velutina* | 13 (15.3%) | 12 (14.1%) | 0 | 2 (15.4%) |
| *Q. coccinea/falcata* | 4 (10.5%) | 6 (15.8%) | 2 (28.6%) | 2 (28.6%) |
|  | **Mother outside** | | | |
| All species | 8 (3.7%) | 43 (19.6%) | 38 (21.2%) | 32 (17.9%) |
| *Q. rubra* | 7 (7.3%) | 15 (15.6%) | 35 (22%) | 25 (15.7%) |
| *Q. velutina* | 0 | 11 (12.9%) | 0 | 6 (46.2%) |
| *Q. coccinea/falcata* | 1 (2.6%) | 17 (44.7%) | 3 (42.9%) | 1 (14.3%) |
|  | **Father outside** | | | |
| All species | 73 (33.3%) | 60 (27.4%) | 70 (39.1%) | 92 (51.1%) |
| *Q. rubra* | 33 (34.4%) | 24 (25%) | 67 (42.1%) | 84 (52.8%) |
| *Q. velutina* | 32 (37.6%) | 29 (34.1%) | 2 (15.3%) | 4 (30.8%) |
| *Q. coccinea/falcata* | 8 (2.1%) | 7 (18.4%) | 1 (14.3%) | 4 (57.1%) |
|  | **Both parents outside** | | | |
| All species | 97 (44.3%) | 81 (37%) | 43 (24%) | 14 (7.8%) |
| *Q. rubra* | 32 (33.3%) | 40 (41.7%) | 31 (19.5%) | 13 (8.2%) |
| *Q. velutina* | 40 (47.1%) | 33 (38.8%) | 11 (84.6%) | 1 (7.7%) |
| *Q. coccinea/falcata* | 25 (65.8%) | 8 (21%) | 1 (14.3%) | 0 |
